# The rs1990760 polymorphism within the IFIH1 locus is not associated with Graves' disease, Hashimoto's thyroiditis and Addison's disease

**DOI:** 10.1186/1471-2350-10-126

**Published:** 2009-12-04

**Authors:** Marissa Penna-Martinez, Elizabeth Ramos-Lopez, Inka Robbers, Heinrich Kahles, Stefanie Hahner, Holger Willenberg, Nicole Reisch, Christian Seidl, Maria Segni, Klaus Badenhoop

**Affiliations:** 1 Department of Internal Medicine I, Division of Endocrinology, Diabetes and Metabolism, University Hospital Frankfurt, Frankfurt am Main, Germany; 2 Department of Internal Medicine I, Division of Endocrinology and Diabetes, University Hospital Wuerzburg, Wuerzburg, Germany; 3 Department of Endocrinology, Diabetes and Rheumatology, University Hospital Duesseldorf, Duesseldorf, Germany; 4 Department of Internal Medicine, Division of Endocrinology and Diabetes, University Hospital Munich, Munich, Germany; 5 Institute of Transfusion Medicine and Immunohaematology, Department of Transplantation Immunology, Red Cross Blood Donor Service, Frankfurt am Main, Germany; 6 Department of Pediatrics Endocrinology Unit, University "La Sapienza," Rome, Italy

## Abstract

**Background:**

Three genes have been confirmed as major joint susceptibility genes for endocrine autoimmune disease:human leukocyte antigen class II, cytotoxic T-lymphocyte antigen 4 and protein tyrosine phosphatase non-receptor type 22. Recent studies showed that a genetic variation within the interferon induced helicase domain 1 (IFIH1) locus (rs1990760 polymorphism) is an additional risk factor in type 1 diabetes and Graves' disease (GD).

**Methods:**

The aim of the present study was to investigate the role of the rs1990760 polymorphism within the IFIH1 gene in German patients with GD (n = 258), Hashimoto's thyroiditis (HT, n = 106), Addison's disease (AD, n = 195) and healthy controls (HC, n = 227) as well as in 55 GD families (165 individuals, German) and 100 HT families (300 individuals, Italian). Furthermore, the interaction between rs1990760 polymorphism with human leukocyte antigen (HLA) risk haplotype DQ2(DQA*0501-DQB*0201), the risk haplotypes DQ2/DQ8 (DQA*0301-DQB*0302) and the status of thyroglobulin antibody (TgAb), thyroid peroxidase antibody (TPOAb) and TSH receptor antibody (TRAb) in patients and families were analysed.

**Results:**

No significant differences were found between the allele and genotype frequencies for rs1990760 IFIH1 polymorphism in patients with GD, HT, AD and HC. Also no differences were observed when stratifying the IFIH1 rs1990760 polymorphism for gender, presence or absence of thyroid antibodies (GD:TRAb and HT:TPOAb/TgAb) and HLA risk haplotypes (DQ2:for GD and HT, DQ2/DQ8:for AD). Furthermore the transmission analysis in GD and HT families revealed no differences in alleles transmission for rs1990760 IFIH1 from parents with or without HLA risk haplotype DQ2 to the affected offspring. In contrast, by dividing the HT parents according to the presence or absence of thyroid Ab titers, mothers and fathers both positive for TPOAb/TgAb overtransmitted the allele A of IFIH1 rs1990760 to their HT affected offspring (61.8% vs 38.2%;p = 0.05;corrected p [pc] = 0.1). However, these associations did not remain statistically significant after correction of the p-values.

**Conclusion:**

In conclusion, our data suggest, no contribution from IFIH1 rs1990760 polymorphism to the pathogenesis of either Graves' disease, Hashimoto's thyroiditis or Addison's disease in our study populations. However, in order to exclude a possible influence of the studied polymorphism in specified subgroups within patients with autoimmune thyroid disease, further investigations in larger populations are needed.

## Background

Autoimmune diseases are characterised by the failure to maintain self-tolerance, which results from the interaction between genetic and enviromental factors. The Major Histocompatibility Complex, the cytotoxic T lymphocyte antigen 4 locus and the protein tyrosine phosphatase non-receptor 22 gene are involved in the pathogenesis of autoimmune endocrine diseases [1]. Recently, a novel locus called Interferon-induced helicard (IFIH1), rs1990760, was identified to be associated with type 1 diabetes (T1D) and Graves' disease [2,3]. IFIH1 gene, also referred to as melanoma differentiation-associated 5 or Helicard, is located on the chromosome 2q24 and encodes an early type I interferon beta responsive gene. The helicase detects cytoplasmatic viral double strand RNA (dsRNA), leading to interferon beta production via activation of interferon regulatory factor-3 (IRF-3) and nuclear factor kappa-B (NFkB). The inappropriate activation of these virus-sensitive proteins by self-derived, intracellular nucleic acids might have an effect in autoimmunity [4]. Therefore and in order to determine whether IFIH1 rs1990760 polymorphism is involved in the genetic background of other endocrine autoimmune disease we compared genotype and allele frequencies in German patients with Graves' disease (GD), Hashimoto's thyroiditis (HT), Addison's disease (AD) and healthy controls (HC) and performed transmission disequilibrium tests (TDT) for that polymorphism in families with GD (German) and families with HT (Italian). Furthermore, we analyzed the distribution of IFIH1 rs1990760 polymorphism in families and case-controls according to the presence or absence of the human leukocyte antigen (HLA) risk haplotype DQ2 (DQA*0501-DQB*0201), risk haploypes DQ2/DQ8 (DQA*0301-DQB*0302)] and thyroid antibodies status.

## Methods

### Subjects

Patients of German origin with Graves' disease (n = 258, 207 females and 51 males), Hashimoto's thyroiditis (n = 106, 87 females and 19 males), Addison's disease (n = 195, 138 females and 57 males) and 55 GD families comprising both parents and a single affected offspring (165 subjects) were recruited from the endocrine outpatient clinics at the University Hospitals of Frankfurt am Main, Freiburg, Wuerzburg, Duesseldorf and Munich, Germany. Healthy controls (n = 227, 160 females and 67 males) were volunteer blood donors from the staff personnel or medical students from the University Hospital Frankfurt am Main, Germany, without a family story of autoimmune diseases. The median age of the patients with GD, HT, AD and HC was 45 (range 7-80), 37 (range 22-70), 51 (range 19-87) and 42 (range 30-101) years respectively. The median age of affected offspring from the 55 GD families was 43 (range 26-55) years. Additionally,100 Hashimoto's thyroiditis families (300 subjects) were recruited from the Pediatrics Endocrinology University "La Sapienza," Rome, Italy. The median age of affected offspring was 11 (range 2-21) years.

Graves' disease diagnosis rested on autoimmune hyperthyroidism with TSH receptor antibodies and/or ophthalmopathy. Hashimoto's thyroiditis was diagnosed by positive thyroglobulin (Tg) and/or thyroid peroxidase antibodies, reduced echogenicity on thyroid ultrasound, and normal or elevated thyrotropin (TSH) levels. Addison's disease was diagnosed by primary adrenocortical insufficiency without evidence of tuberculosis or adrenoleucodystronphy. The study protocol was approved by the ethics committee of the University Hospital Frankfurt am Main, Germany and the University "La Sapienza," Rome, Italy. A informed consent was obtained from all participants.

### Genotype analysis

Blood samples were collected in ethylenediamine tetraacetic acid-anticoagulated tubes from study participants and genomic DNA was extracted using salting out procedure [5]. The samples were genotyped for IFIH1 rs1990760 polymorphism by using Taqman assay C__2780299_30 in a ABI 7300 real-time PCR system under the conditions recommended by the manufacturer (Applied Biosystems, Darmstadt, Germany). The HLA DQA1 and DQB1 typing was performed as described previously [6]. In order to confirm the accuracy of the methods, random samples of the IFIH1 rs1990760 polymorphism and HLA were genotyped twice with a concordance of 98%.

#### Measurement of TPO-, TG- and TRAb antibodies

Thyroglobulin antibody (TgAb) and thyroid peroxidase antibody (TPOAb) were measured by enzyme-linked Immunosorbent Assay (Phadia, Freiburg, Germany) and TSH receptor antibodies (TRAb) were determined by TRAK Assay kit (Brahms, Berlin, Germany).

### Statistical analysis

Patients and controls and their subgroups (see results) were compared using allele-wise and genotype-wise Chi^2 ^testing by using *BiAS software *(package 8.21, Epsilon, Weinheim, Germany). Family analyses were estimated using *Haploview software *version 3.2 available from http://www.broad.mit.edu/mpg/haploview to detect preferential transmission of the IFIH1 rs1990760 alleles to the affected subjects, whereas the correlation with HLA haplotypes [risk haplotype: DQ2 (DQA*0501-DQB*0201) for GD and HT and risk haplotypes DQ2/DQ8 (DQA*0301-DQB*0302) for AD and non risk haplotypes (neither DQ2, neither DQ2/DQ8, respectively)] and thyroid antibodies (ThyAbs) was evaluated using *Unphased *software version 2.403 available from http://www.mrc-bsu.cam.ac.uk/personal/frank/software/unphased/. All p values were corrected (Bonferroni correction) by multiplication with the number of alleles tested (× 2). A corrected p (pc) < 0.05 was considered significant.

## Results

Allele and genotype frequencies for IFIH1 rs1990760 polymorphism in all subjects did not deviate from Hardy-Weinberg equilibrium (HWE; p > 0.05).

No significant differences were found between the allele and genotype frequencies for IFIH1 rs1990760 polymorphism in patients with GD, HT, AD and HC (Table 1). Also no differences were observed when stratifying the rs1990760 IFIH1 polymorphisms according to gender in each patient groups. Furthermore, GD and HT patient groups were divided in HLA risk haplotype (DQ2) and HLA non risk haplotype (neither DQ2), the AD patients in HLA risk haplotypes (DQ2/DQ8) and non risk haplotypes (neither DQ2/DQ8). Additionally, following other subgroups were analysed a) GD: group TRAb positive (pos)/group TRAb negative (neg), b) HT: group TPOAb pos/group TPOAb neg, c) HT: group TgAb pos/group TgAb neg. Next all measured groups were stratified for gender. However, the comparison between the patient subgroups and their corresponding control groups did not reach the level of significance (data not shown).

**Table 1:** Genotype and allele frequencies for IFIH1 rs1990760 polymorphism in German patients with Graves'disease (GD), Hashimoto's thyroiditis (HT), Addison' s disease (AD) and healthy controls (HC)

	HD (n = 227)		GD (n = 258)				HT (n = 106)				AD (n = 195)			
										
	n	%	n	%	p	pc	n	%	p	pc	n	%	p	pc
Genotype														
GG	28	12.3	38	14.7			20	18.9			28	14.4		
GA	112	49.3	132	51.2	0.55	1.1	45	42.5	0.23	0.46	99	50.8	0.7	1.4
AA	87	38.3	88	31.1			41	38.7			68	34.9		

Allele														
G	168	37	208	40.3	0.32	0.64	85	40.1	0.49	0.98	155	39.7	0.45	0.9
A	286	63	308	59.7			127	59.9			235	60.3		

pc = p corrected

The transmission analysis for IFIH1 rs1990760 polymorphism in 100 HT families (Italy) revealed no differences. In order to evalute the effect of ThyAb on the IFIH1 rs1990760 polymorphism, the parents were divided into four subgroups according to their TPOAb/TgAb titers: 39 families, no ThyAb were detectable in either mothers or fathers (group 1), 35 families, only mothers had detectable antibodies (group 2), 9 families, only fathers were positive for antibodies (group 3), and 17 families, both parents presented detectable antibodies (group 4). The analysis showed that mothers and fathers in group 4 overtransmitted the allele A of rs1990760 IFIH1 to their HT affected offspring (61.8% vs 38.2%; p = 0.05; corrected p [pc] = 0.1, Table 2).

**Table 2:** Transmission (T) and non transmission (NT) of IFIH1 rs1990760 polymorphism in 100 Hashimoto's thyroiditis families (Italian) according to the presence of thyroid antibodies (TPO and TG)

Polymorphism	Allele	Frequency	T		NT		p	pc
rs1990760		(%)	n	(%)	n	(%)		
Group	A	55.5	110	(49.5)	112	(50.5)	0.84	1.68
100 parents	G	44.5	90	(50.6)	88	(49.4)		

Group 1	A	53.2	38	(45.8)	45	(54.2)	0.26	0.52
39 mo-, fa-	G	46.8	40	(54.8)	33	(45.2)		

Group 2	A	57.9	41	(50.6)	40	(49.4)	0.86	1.72
35 mo+, fa-	G	42.1	29	(49.2)	30	(50.8)		

Group 3	A	66.7	10	(41.7)	14	(59.3)	0.15	0.3
9 mo-, fa+	G	33.3	8	(66.7)	4	(33.3)		

Group 4	A	50.0	21	(61.8)	13	(38.2)	0.05*^1^	0.1
17 mo+, fa+	G	50.0	13	(38.2)	21	(61.8)		

mo = mothers; fa = fathers

pc = p corrected

*^1^OR = odds ratio; (95% CI) = 95% confidence interval.

group 4: allele A OR 2.61 (0.98-6.94) and allele G OR 0.38 (0.14-10.2)

p = 0.05

However, the associations obtained in the family analyses did not remain statistically significant after correction of the p-values. Because TRAb titers of the parents in the GD families were not available, the transmission analysis was not performed. Additionally, we compared the transmission of the IFIH1 rs1990760 polymorphism in parents, in either mothers and fathers in GD and HT families with the haplotype DQ2 and without DQ2 by TDT. In Italian HT families as well as the in German GD families the interaction analysis between IFIH1 rs1990760 polymorphism and HLA DQ2 haplotype revealed no difference in the transmission (data not shown).

## Discussion

The innate immune system detects dsRNA, a replication product of most viruses, within infected cells by Toll-like receptor 3 and cytoplasmic RNA helicases such as retinoic acid inducible gene I and interferon-induced helicase. IFIH1, a 1025 amino acid cytoplasmic protein, protects the host from viral infection by triggering a cellular antiviral and apoptotic response. In this process IFIH1 signaling activates transcription factors, IRFs and NFkB pathways through its caspase recruitment domain (CARD) that induce IFN-α/β and interferon-inducible genes [7-9]. IFIH1 transcripts were found to be expressed in lymphoid and other tissues, suggesting a role in autoimmune diseases. A large scale association study of candidate (nonsynonymous, i.e. amino acid changing) polymorphism identified IFIH1, rs1990760, as a new locus for T1D [2]. The rs1990760 polymorphism is located in HNF-3b binding site within exon 15, encodes an alanine to threonine amino acid change at codon 946 (Ala946Thr), which does not reside in either the CARD or helicase domain of the protein. However, this region of the protein is conserved between mammals and may have other, unknown functions or may influence the active domains through effects on tertiary structure. Ala946 is the fully conserved amino acid, suggesting that this is the functional allele. The association between IFIH1 rs1990760 polymorphism and T1D was not only confirmed by other authors [10-12] but also reported in other autoimmune diseases [3,13,14]. Studies that reported associations between viral infection and susceptibility to development T1D as well as autoimmune thyroid disease (ATD) [15-17], reinforce the IFIH1 gene as a good functional candidate for these autoimmune disorders. On the basis on this finding we investigated the role of IFIH1 rs1990760 polymorphism in ATD including GD, HT and Addison's disease (all Germans) and enlarge our analysis in the transmission of this polymorphism in families with offspring affected with GD and HT. Similar to other studies, that investigated IFIH1 rs1990760 polymorphism in diverse autoimmune disease we could not find a association between IFIH1 rs1990760 polymorphism and our case-control study population [18,19]. In contrast, Sutherland et al. (2007) tested the same coding variant for association to ATD (646 cases;446 controls) and autoimmune Addison's disease (204 cases) from Caucasian United Kingdom (UK) and found that the A allele increased the odds of Graves' disease 1.47-fold. Since in our study the distribution of rs1990760 polymorphism in controls (Germans) was not different from that in UK controls and the fact that Sutherland et al. (2007) analogue to our findings did not detect an association in AD patients, the smaller number of patients could be an explanation for the lack of an association between endocrine autoimmune diseases and the rs1990760 polymorphism observed in the present study. However, the trend associations obtained in the HT family in which mothers and fathers were both positive for TPOAb/TgAb, overtransmitted the allele A of IFIH1 rs1990760 to their HT affected offspring (61.8% vs 38.2%;p = 0.05; corrected p [pc] = 0.1), did not remain statistically significant after correction of the p-values. This may reflect that the selection of the patient collectives could influence the detection of an association.

Also the rare polymorphisms, rs35667974 and rs35337543 which reside within the IFIH1 gene were found to be associated with T1D [20] but their effect can only be studied in large population samples. Nevertheless these data point to a possible role of IFIH1 in the pathogenesis of T1D and marke the investigations of large cohorts necessary.

The IFIH1 rs1990760 polymorphism is in linkage disequilibrium (LD) with other IFIH1 variants for instance rs2068330, rs2111485 and rs984971 and could be different due to population heterogeneity [12]. Therefore the different patterns of LD across studies and populations could be a further explanation for the discrepancy of the results.

## Conclusion

We conclude from the results of the present case-control and families study that there is no significant role of the IFIH1 rs1990760 polymorphism in the genetic susceptibility to GD, HT and AD in Germans and HT in Italians. However in order to exclude a possible influence of the rs1990760 polymorphism in specified subgroups within patients with ATD further investigations in larger samples are required. Also other polymorphisms e.g. rs2068330, rs2111485, rs984971 within the IFIH1 LD block are recommended to be investigated in future studies, in order to obtain more suitable markers to analyse the susceptibility for the development ATD and AD in Germans.

## Abbreviations

IFIH: Interferon-induced helicard; dsRNA: double strand RNA; IRF: interferon regulatory factor; NFkB: nuclear factor kappa-B; GD: Graves'disease; HT: Hashimoto's thyroiditis; AD: Addison's disease; HC: healthy controls; TDT: transmission disequilibrium tests; HLA: human leukocyte antigen; CARD: caspase recruitment domain; T1D: type 1 diabetes; ATD: autoimmune thyroid disease; UK: United Kingdom.

## Competing interests

The authors declare that they have no competing interests.

## Authors' contributions

MPM, ERL and IR carried out the genotyping of the patients and controls. HK participated in the statistical analysis. SH, HW, NR, CS, MS made the diagnosis and collaborated in the collection of samples. MPM and KB conceptualized and designed the study. All authors read and approved the manuscript.

## Pre-publication history

The pre-publication history for this paper can be accessed here:

http://www.biomedcentral.com/1471-2350/10/126/prepub
